# InAs Nanowire-Based Twin Electrical Sensors Enabling Simultaneous Gas Detection

**DOI:** 10.1021/acsanm.4c07238

**Published:** 2025-05-08

**Authors:** Camilla Baratto, Egit Musaev, Valeria Demontis, Stefano Luin, Valentina Zannier, Lucia Sorba, Guido Faglia, Luigi Rovati, Francesco Rossella

**Affiliations:** † CNR-INO PRISM Lab, Via Branze 45, Brescia 25123, Italy; ‡ Department of Information Engineering, University of Brescia, Via Branze 38, Brescia 25123, Italy; § Department of Physics, University of Cagliari, S.P. Monserrato-Sestu, Monserrato 09042, Italy; ∥ NEST Laboratory, Scuola Normale Superiore and Institute of Nanoscience - CNR, piazza San Silvestro 12, Pisa 56127, Italy; ⊥ Department of Engineering “Enzo Ferrari”, University of Modena and Reggio Emilia, Via Vivarelli, 10, building 26, Modena I-41125, Italy; # Dipartimento di Scienze Fisiche, Informatiche e Matematiche, Università di Modena e Reggio Emilia, Via Campi 213/a, Modena I-41125, Italy

**Keywords:** InAs nanowires, gas detection, nanosensors, signal fluctuations, electrical transport

## Abstract

Epitaxially grown InAs NWs are relevant for electrical sensing applications due to the Fermi level pinning at the NW surface and are highly sensitive to the surrounding environment. While a single NW growth batch consists of millions of virtually identical replicas of the same NW, real samples display subtle differences in NW size, shape, and structure, which may affect detection performance. Here, electrical gas detection is investigated in two nominally identical or twin devices fabricated starting from the same NW growth batch. Two individual wurtzite InAs NWs are placed onto a fabrication substrate at a 2 μm distance with a 90° relative orientation, each NW is electrically contacted, and the nanodevices are exposed to humidity and NO_2_ flux diluted in synthetic air. Electrical signal versus time is measured simultaneously in each nanodevice upon exposure to different gases and concentrations. The observed detection limit is 2 ppm for NO_2_ and 20% for relative humidity. Correlation analysis methods are exploited by calculating autocorrelation and cross-correlation functions for the experimental signal pairs, indicating lack of cross-correlation in the signal noise of the two nanodevices, suggesting that signal differences between the twins could be ascribed mainly to nonidealities in the fabrication protocol and nanoscopic differences in the two nanostructures, rather than to different environmental conditions. While InAs nanowires are used here as demonstrators of simultaneous gas sensing, the approach is general and virtually applies to any nanoscale material suitable for the realization of two-terminal electronic devices.

## Graphical abstract

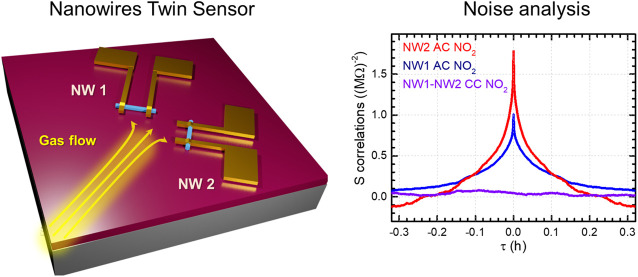

## Introduction

Sensors have become essential devices in many modern technologies, driving the quest for low-cost, easily addressable solutions for several applications, especially environmental gas detection.^1^ The advancement of nanotechnology and nanofabrication techniques has enabled the development of novel and advanced nanodevices for sensing applications, based on nanostructures such as semiconductor nanowires (NWs).^2^ Multiple types of sensors based on individual semiconductor NWs or NW arrays^3–5^ using both optical and electrical signal transduction principles have been largely demonstrated.^6–9^ Recent studies on nanowire-based devices enabling simultaneous measurements have shown significant progress toward the integration of multifunctional sensors into soft robotics^10^ and textiles^11,12^ as well as for precise gas detection applications.^13^

Conductometric sensors, based on current modulations induced by surface interactions between gases and the electrical conductor, stand out as cost-effective and reliable options. In a gaseous environment, sensing based on semiconductors is promoted either by charge transfer to or from adsorbed molecules or by gas-induced alteration of the height of Schottky barriers generated at the metal–semiconductor contacts.^14,15^ NWs are especially suitable for applications in gas sensing because they maximize the surface-to-volume ratio, allowing for high sensitivity, and display well-defined crystalline facets, ensuring stability (e.g., over time). In fact, effective sensors exploiting direct electrical readout have been engineered by using semiconductor NWs,^16–19^ and in general, these systems are considered to have huge potential for next-generation chemical sensors.

On the one hand, a family of experimental studies focuses on the performance of ensembles of NWs contacted by two global electrodes.^20,21^ By using this approach, the estimation of the electrical properties of NWs, and consequently the device performances, is mediated among all the nanostructures composing the array, although the individual nano-objects can differ from one another . The macroscopic contacts connect, ideally in parallel, thousands of NWs, although the global electrical properties are affected by the potential barrier between two or more different NWs touching each other^22^ like in nanostructured films, where grain boundaries govern the conduction. On the other hand, single NW-based sensors are regarded as greatly promising for specific applications. For instance, in the case of metal oxide-based gas sensors such as SnO_2_ and ZnO, fundamental studies on the behavior of the single NW have enabled a quite deep comprehension of the sensing mechanism.^23–25^ However, metal oxide-based sensors have the drawbacks of high-temperature operation^26^ and reliance on UV-induced adsorption/desorption phenomena for their room-temperature operation.^27^

Epitaxially grown InAs NWs are regarded as a very promising nanomaterial platform for the development of gas sensors operating at room temperature. In these nanostructures, the occurrence of Fermi level pinning at the surface promotes the presence of high-density surface states and the onset of an accumulation layer, which can be extremely useful for sensing the environment surrounding the NW surface, particularly for detecting the presence of gases that can be absorbed as charged molecules.^28^ InAs NWs in array configurations have been exploited for ethanol^29^ as well as NO_2_ ^30^ detection at very low concentrations, diluted in a N_2_ atmosphere. Regarding individual InAs NW-based sensors, they have been reported to be suitable for humidity and organic vapor detection in an inert atmosphere (either nitrogen or helium),^28,31,32^ and different device configurations have been investigated.^6^ Naively, all of the NWs isolated from the same growth batch can be regarded as identical replicas of the same ideal structure. However, in real samples, differences between the NWs are unavoidable, and depending on the targeted applications, such differences may play a minor role or represent a major drawback. For instance, NW-to-NW property variations may result in small changes in the electrical response of devices fabricated starting from different NWs. Moreover, the same nanodevice fabrication protocol applied to identical NWs may lead to slightly different devices. Therefore, an open issue regarding any type of electrical sensors developed starting from single nano-objects, such as single InAs NWs, is the dependence of the device response upon the characteristics of the specific nanostructure, namely, crystalline purity, morphology, and dimensions. Moreover, the specific device features, namely nanostructure orientation with respect to electrodes, as well as electrode materials, dimensions, and thickness, may affect the detection performance. Indeed, all the potential differences between two ideally identical nanoscale sensors represent in turn potential sources of differences in the response signals of the two devices and may induce noise in the measurements.

In this work, we report the design, realization, and experimental measurement, at room temperature, of two InAs NW-based nominally identical (twin) devices operating as electrical sensors of gases. The two NW-based sensors were fabricated starting from nanostructures with a diameter difference not exceeding 10 nm isolated from the same growth batch, and the architecture was designed in order to ensure that the two nanowires were exposed to the gas flow in the same conditions. Specifically, the two devices were placed at approximately 2 μm distance with a 90-degree relative orientation. A custom readout interface was developed to enable simultaneous measurement of the two devices during the sensing experiments. Based on the experimental outcomes, we carried out signal correlation analysis by calculating autocorrelation and cross-correlation functions of the measured electrical current signal noise (estimated from fit residuals upon device exposure to different gases with varying concentrations. We found an overall lack of cross-correlation, on the time scale of seconds, in the electrical noise response of the two nanodevices measured under the same conditions. The different fluctuations in the responses of the two detectors are not attributable to different environments surrounding the surfaces of the two NWs locally but instead to unidealities in the fabrication protocol combined with slight (nanoscopic) differences in the individual nanostructures and their interaction with gas molecules

Here, InAs nanowires are used as demonstrators due to their ability in the conductometric sensing of the surrounding environment, which is associated with the Fermi level pinning at the surface, combined with their high aspect ratio and the ease of fabricating ohmic contacts for engineering nanoscale electrical devices. However, it is worth highlighting the full generality of the approach presented in this work, which can be virtually applied to any nanomaterial suitable for the realization of two-terminal electronic devices.

Overall, the novelty added to the field of nanoscale conductometric sensing by the present work is manifold. It addresses the potential impact of individual nanoscale device features on the sensing performance of nanoelectronic gas detectors by exploiting individual InAs nanowires grown by chemical beam epitaxy. Additionally, the methodology is new: pairs of virtually identical nanowire-based devices are engineered and exposed to nominally identical gas environments, the electrical response of the two devices is measured, and signal fluctuation analysis is exploited to identify the differences in the two simultaneous signals. Importantly, this methodology applies virtually to any nanoscale object suitable for the realization of two-terminal electronic devices.

## Experimental Section

### Nanowire Growth and Device Nanofabrication

Wurtzite InAs NWs were grown by gold-assisted chemical beam epitaxy (CBE) on InAs (111)B substrates, using trimethylindium (TMIn) and tertiarybutylarsine (TBAs) metallorganic precursors.^33^ Gold nanoparticle catalysts were obtained by dewetting (at 540 ± 10^°^ C under TBA flow for 20 min) of a 0.5 nm thick gold film, previously evaporated on the InAs substrates. The NWs were grown at a temperature of 465 ± 10° C, with TMIn and TBA line pressures of ≈ 0.9 and ≈ 0.3 Torr, respectively, for a time of ≈ 45 min. Ditertiarybutylselenide (DtBSe) with a line pressure of 0.10 Torr was used as n-doping source. After growth, the NWs were mechanically detached from the growth substrate and dispersed in isopropyl alcohol (IPA) by sonicating the substrate in IPA. A droplet of the IPA/NWs solution was then deposited by drop-casting onto a SiO_2_/SiO_2_^+2^ substrate prepatterned with markers for nanofabrication. The contact electrodes were patterned by a single-step aligned electron beam lithography (EBL). After the development and immediately before the evaporation of a bilayer metal contact (Cr/Au, 10/100 nm), the NW contact areas were exposed to an ammonium polysulfide (NH_4_)_2_S-based solution to promote the formation of low-resistance ohmic contacts. The chip was then attached to a standard dual-in-Line chip carrier using a conductive silver paste, and the devices were wire-bonded to the carrier.

### Customized Electronic Circuitry for Simultaneous Sensing with Two Nanowires

In order to simultaneously measure the electrical response of each NW, we designed a dedicated electronic circuit to feed a constant current through the two NWs and measure the voltage drop across them: driving a constant current allows us to avoid heating-induced changes in the NW response. In fact, self-heating in nanostructures, including NWs, can occur even at very low electrical power.^34^ While this effect is less pronounced in substrate-supported NWs compared to suspended ones,^35^ it must still be considered. To avoid NW damage due to self-heating, a stable current of 2 μA was fed across the nanostructures during the experiments. The circuit also allows for measuring the resistance of the NW twin devices and comprises six key components: two constant current source circuits based on LM334,^36^ a voltage amplifier circuit in the noninverting operational amplifier configuration (Figure 1b) based on AD8656,^37^ an analog-to-digital converter (ADC) based on ADS1220,^38^ an Arduino UNO (MCU) board [35] (Figure 1a), an ambient temperature sensor SHT33-dis to measure the temperature outside the test chamber [36], and a PC. In the circuit (Figure 1b), the resistor element-labeled “NW” corresponds to the device.

**Figure 1:**
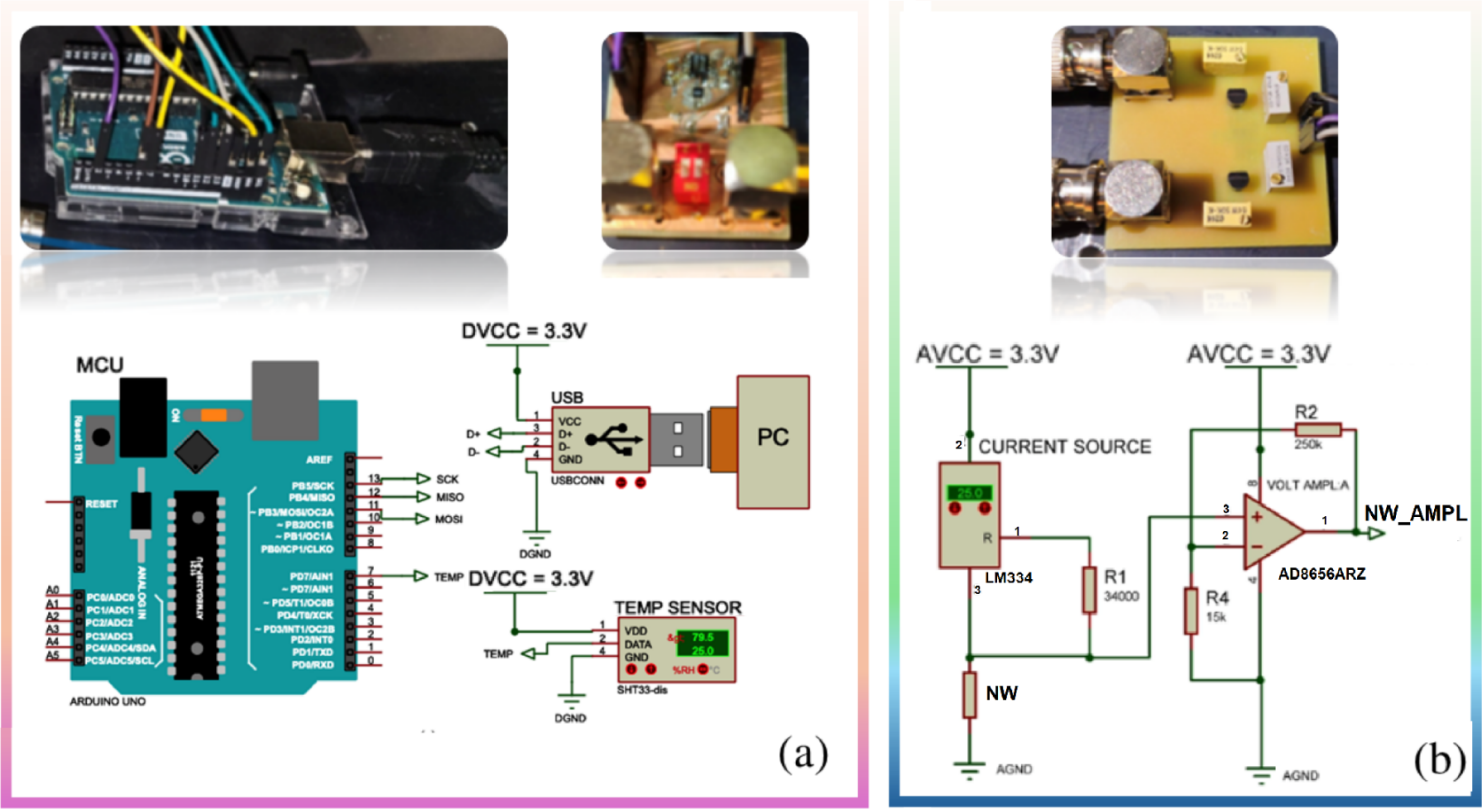
Schematic block diagram and images of (a) data processing and data transmission components; (b) NW resistance voltage conversion circuit (one for each nanowire).

As shown in Figure 1, the current sources are configured to provide a consistent current to the NWs, set at 2 μA, determined by the resistor R1.^36^ As the current flows through the NWs, it generates a voltage drop according to Ohm’s law. The voltage signal of each NW is then amplified by the voltage amplifier with a gain factor of 16.5. Consequently, when the resistance of the NWs reaches its peak value of 100 kΩ, the resultant voltage is approximately 3.5 V. This value represents the maximum input voltage for the ADC, ensuring optimal resolution for current measurements. The ADC transmits information regarding the voltage drop observed across the NWs to the MCU via a serial peripheral interface. The MCU then processes these data. To account for the temperature drift of the current source, a temperature compensation mechanism was integrated into the circuit design. This mechanism comprises a temperature sensor that is capable of measuring the temperature around the current source circuit and transmitting this information to the MCU for processing. By taking into account the data obtained from the ADC and the temperature sensor, the MCU computes the true value of the resistance of the NWs, thereby correcting for any temperature-induced variations in the current source output. In real time, the PC retrieves data on the resistance of the NWs via a USB interface connected to the MCU. This information is then visualized and concurrently saved into text files, facilitating subsequent analysis.

The Arduino unit serves as a galvanically isolated interface between the measurement board and the PC, ensuring no electrical coupling that could influence signal integrity. The measurement board‘s noise is caused by the current source, amplifier, and analog-to-digital converter (ADC), and detailed noise calculations for each component are reported later in Subsection “Noise calculation”. When designing the overall measurement system, we targeted a noise level equivalent or slightly higher than the one reported for Keithley digital multimeters of the 2400 series, in particular 0.5 × 10^–4^ % for a 2 μA current, where such a current level is consistent with the bias currents applied to the InAs nanowire devices used in this work. As a matter of fact, the noise of our measurement system was estimated to be in the range of 2–5 × 10^–4^ % for a nanowire-based device with an electrical resistance of 100 kΩ, comparable to the noise of digital electrometers and considerably lower than the noise experimentally detected in the twin nanodevices.

### Gas Sensor Testing Setup

Sensing tests were carried out in a cylindrical stainless steel test chamber (300 cm^3^) with a constant flux of synthetic air (100 cm^3^/min). The ratio between gas flux and test chamber size was chosen to avoid turbulence inside the chamber, at the expense of the chamber filling time, which should be at least greater than 3 min. Gas flow enters from the top of the chamber, and the exhaust is placed at the bottom of the chamber. NO_2_ at ppm concentration is obtained from a commercial bottle, while humid air is generated using the method referred to as “flow through”.^39^

### Autocorrelation and Crosscorrelation Functions

The autocorrelations (ACs) *G*_11_(0) and *G*_22_(0) at time τ = 0 correspond to the variances of two signals ″1″ and ″2″, and the ACs *G*_11_(τ) and *G*_22_(τ) at lag time τ≠ 0 and the cross-correlations (CCs) *G*_12_(τ) (= *G*_21_(-τ)) at lag time τ correspond to the covariances of the signals at such time lag, evaluated over the populations of measurements taken at different times *t*. The time scales of the fluctuations (the ″noise″) can be inferred from the time behavior of the AC function, and these are related to the time scales of the system for reaching equilibrium (fluctuation-dissipation theorem).

1 $$r_{12}\left(\tau \right)=\frac{G_{12}\left(\tau \right)}{\sqrt{G_{11}\left(\tau \right)G_{22}\left(\tau \right)}}$$

The ratio gives a measure of how much two signals are correlated at the time scale τ, similar to the *R* coefficient in regression analyses or the Pearson coefficient in colocalization analyses.^40,41^ Values of *r*_12_(τ) sufficiently close to 1 in noise analyses imply that the fluctuations have the same physical origin for the two signals.

Crosscorrelation analyses have been used in noise analysis to understand the correlations among noise sources in different measurements.^42,43^ They have also been widely employed for studying, through fluorescence crosscorrelation spectroscopy (F­(C)­CS), the dynamics of fluorophores or fluorescently labeled molecules, whether due to translational motions or intrinsic changes caused by the photophysics of the fluorophores). For example, a high crosscorrelation (values of *r*_12_(τ) close to 1) in FCCS implies that the same single molecules or aggregates enter and exit the observation volume(s) (an aggregate or molecule containing two fluorophores for two-color FCCS, or a single molecule moving from one volume to another for spatial FCCS).^44^ In our case, the reported AC and CC functions have been calculated, according to the equations provided in the main text, using homemade scripts and functions in MATLAB R2017b, based on the built-in function xcorr with the ″unbiased″ normalization option. We present the nonnormalized correlation functions in the graphs so that the variances of the signals can be appreciated for τ = 0.

### Noise Calculation

#### LM334 Current Source Noise

**Mechanism**: Current noise density (*I*_noise_) and drift.

**Assumptions**:

Current noise density: $1\mathrm{pA}/\sqrt{\mathrm{Hz}}$ (datasheet-dependent).

Bandwidth: 20 Hz.

**Calculation**:

$$I_{\mathrm{noise}}=1\mathrm{pA}/\sqrt{\mathrm{Hz}}\times \sqrt{20}\approx 4.47\mathrm{pA}$$

Voltage noise across NW (*R* = 100 kΩ):

$$V_{\mathrm{source}}=4.47\mathrm{pA}\times 100k\Omega =0.45\mu V\left(\mathrm{input‐referred}\right)$$

After amplification:

$$V_{\mathrm{source},\mathrm{out}}=0.45\mu V\times 16.5\approx 7.43\mu V$$

**Impact**: Dominates if $i_{\mathrm{noise}}>1\mathrm{pA}/\sqrt{\mathrm{Hz}}$ .

#### AD8656 Amplifier Noise

**Contributions**:

Voltage noise: $10\mathrm{nV}/\sqrt{\mathrm{Hz}}$ (datasheet)

Current noise: $0.1\mathrm{pA}/\sqrt{\mathrm{Hz}}$ (negligible for high-impedance sources)

Resistor thermal noise (feedback network *R*_2_/*R*_3_):

$$V_{\mathrm{res}}=\sqrt{4kT\left(R_{2}+R_{3}\right)B}$$

**Calculations**:

Voltage noise:

$$V_{\mathrm{amp}}=10\mathrm{nV}/\sqrt{\mathrm{Hz}}\times \sqrt{20}\times 16.5\approx 0.74\mu V$$

Resistor noise:

**Resistor noise** (e.g., *R*_2_ = 16.5 kΩ, *R*_3_ = 1 kΩ)

Total amplifier noise:

$$V_{\mathrm{amp},\mathrm{total}}=\sqrt{{\left(0.74\right)}^{2}+{\left(0.31\right)}^{2}}\approx 0.80\mu V$$

#### ADS1220 ADC Noise

**Mechanism**: Input-referred noise (datasheet: 0.5 μV RMS at 20 SPS). **Calculation**:

$$V_{\mathrm{adc}}=0.5\mu V$$

#### Total System Noise

Combine all contributions at the ADC input (RMS sum):

$$V_{\mathrm{total}}=\sqrt{V_{\mathrm{nw},\mathrm{out}}^{2}+V_{\mathrm{source},\mathrm{out}}^{2}+V_{\mathrm{amp},\mathrm{total}}^{2}+V_{\mathrm{adc}}^{2}}$$

$$V_{\mathrm{total}}=\sqrt{{\left(2.97\right)}^{2}+{\left(7.43\right)}^{2}+{\left(0.80\right)}^{2}+{\left(0.5\right)}^{2}}\approx 8.0\mu V$$

**Equivalent Resistance Noise**:

$$R_{\mathrm{noise}}=\frac{V_{\mathrm{total}}}{I\times G}=\frac{8.0\mu V}{2\mu A\times 16.5}\approx 0.24\Omega \mathrm{RMS}$$

For *R*_nw_ = 100 kΩ, this corresponds to 0.00024% noise.

## Results and Discussion

### Nanodevices Architecture

For the present work, InAs nanowires with a diameter of 70 ± 5 nm, grown using gold-assisted chemical beam epitaxy (CBE), are isolated from the same growth batch and used for the fabrication of pairs of nanowire-based noinally identical electrical sensors, hereafter referred to as twin sensors or twin devices. Figure 2a shows a pictorial representation of the gold-assisted chemical beam epitaxy (CBE) process, described in detail in the “Nanowire growth and device nanofabrication” subsection of the Experimental section. The top panel displays the starting growth substrate, where the gold nanoparticle catalysts are previously deposited, and the vapor precursor enters the growth chamber. The bottom panel displays a schematic of the grown nanowire sample. NWs are transferred onto a Si^2+^/SiO_2_ fabrication substrate, and devices are realized starting from NW pairs placed approximately 2 μm apart, oriented at 90° relative to each other. Each NW of the pair is equipped with two nominally identical electrical contacts, realized by evaporating two metallic electrodes (610 ± 10 nm wide) on both ends of the NW, at a distance of 1400 ± 5 nm. In our experiments, a gas flux of synthetic air containing the gas analyte (humidity or NO_2_) flows onto the NW twin devices, while electrical current is measured simultaneously in both nanodevices as a function of time. A custom readout interface enables the simultaneous measurement of the two NWs. Importantly, the flow rate is kept constant during the entire measurement and is decreased to a value where gas turbulence or cooling effects can be neglected.

**Figure 2:**
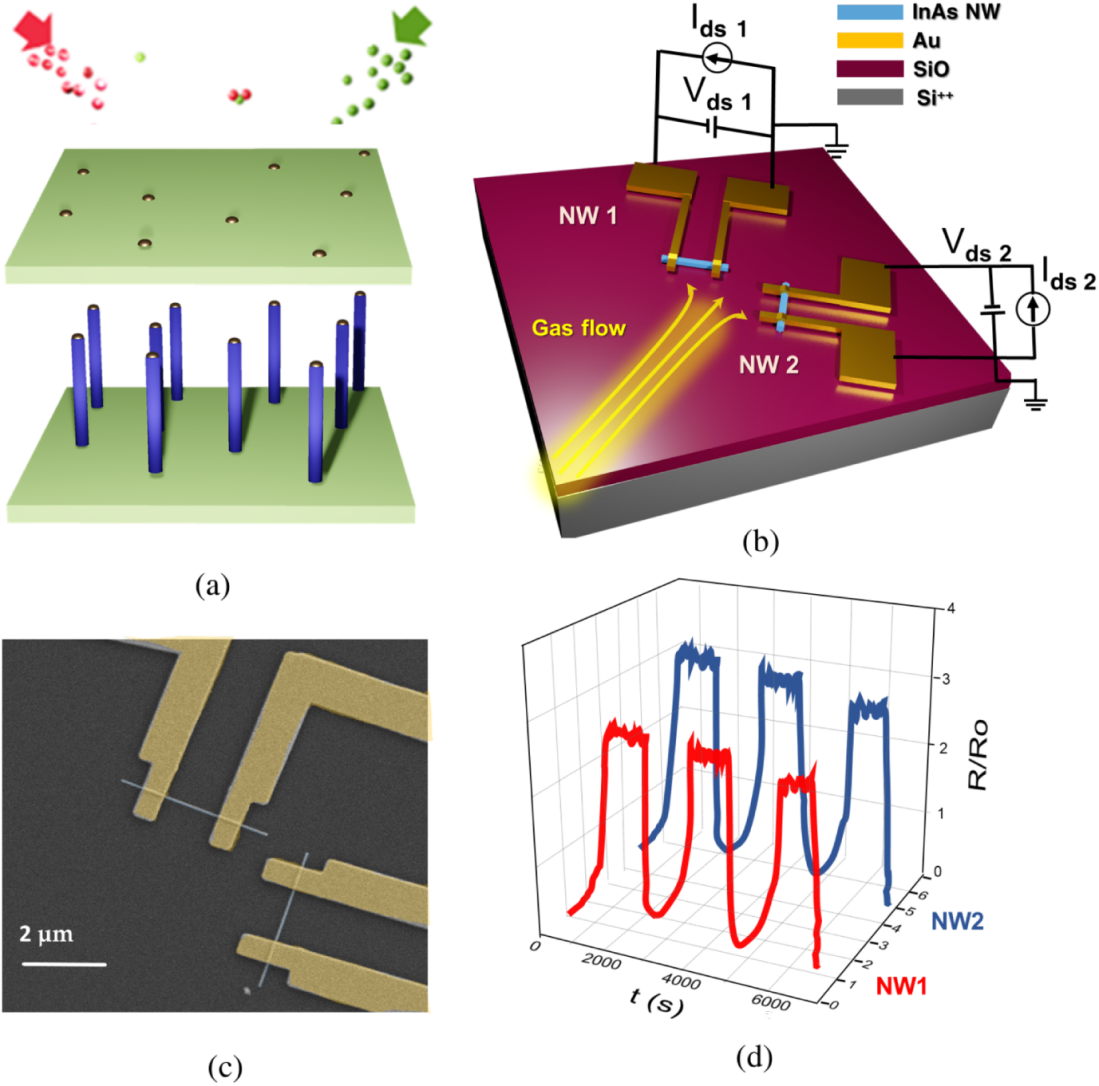
(a) Schematic of the gold-assisted CBE InAs NW growth process, (b) schematic of the two InAs NW-based devices used in this work, (c) scanning electron micrograph of one of the fabricated nanowire twin device, and (d) schematic of the measurement output.

Figure 2b shows a pictorial view of the pair of InAs NW-based devices used in this work, while the false-colored scanning electron micrograph of one of the fabricated twin sensors (top view) is reported in Figure 2c. An example of the measurement output consisting of two resistance-versus-time curves acquired simultaneously in the two NWs is reported in Figure 2d.

Notably, while the difference in diameter between the two nanostructures is likely not exceeding 10 nm, this might yield a fairly significant difference in the surface of the nanostructure exposed to the gas, corresponding to a surface/volume ratio variable in the range from 0.067 (for a 60 nm diameter nanowire) to 0.057 (for a 70 nm diameter nanowire). Developed sensors be very sensitive to the morphological parameters of the nanostructures and the metalized pattern, yielding slight differences in the electric response from device to device.

The purpose of the 90^°^ relative orientation between the twin nanowire devices is illustrated in the pictorial view of the device shown in Figure 2b. In general, the measurement chamber may exhibit a preferential direction in the flow of gas molecules. To ensure that the nanoscale twin device architecture remains resilient to any bias in the gas injection direction, we designed the twin nanodevices with a 90^°^ relative orientation and positioned the chip inside the measurement chamber so that each nanowire is exposed at a 45^°^ angle to the gas flow.

### Simultaneous Gas Detection Measurements

We evaluated the response of the twin NW devices to variations in relative humidity and NO_2_ at room temperature. The parameters used for gas tests, including gas flow rate, working chamber volume, and other details related to the chamber configuration, are provided in the “Experimental Section, Gas Sensors Testing Setup”.

#### RH Detection

We evaluated the twin NW devices’ response to variations of relative humidity at levels ranging from 10% to 70% RH in 10% increment at room temperature. Each testing cycle consisted of a 1 h exposure period during which the sensors were subjected to an input air flow with specific and constant humidity concentration, followed by a 1 h recovery period during which the samples were allowed to recover to their baseline resistance in dry air. The baseline resistance of the NWs typically ranged between 12 and 25 kΩ. The experimental outcomes are presented in Figure 3, which summarizes the performance of the NW twin devices as normalized resistance plotted against time, evidencing remarkable response to humidity exposure.

**Figure 3:**
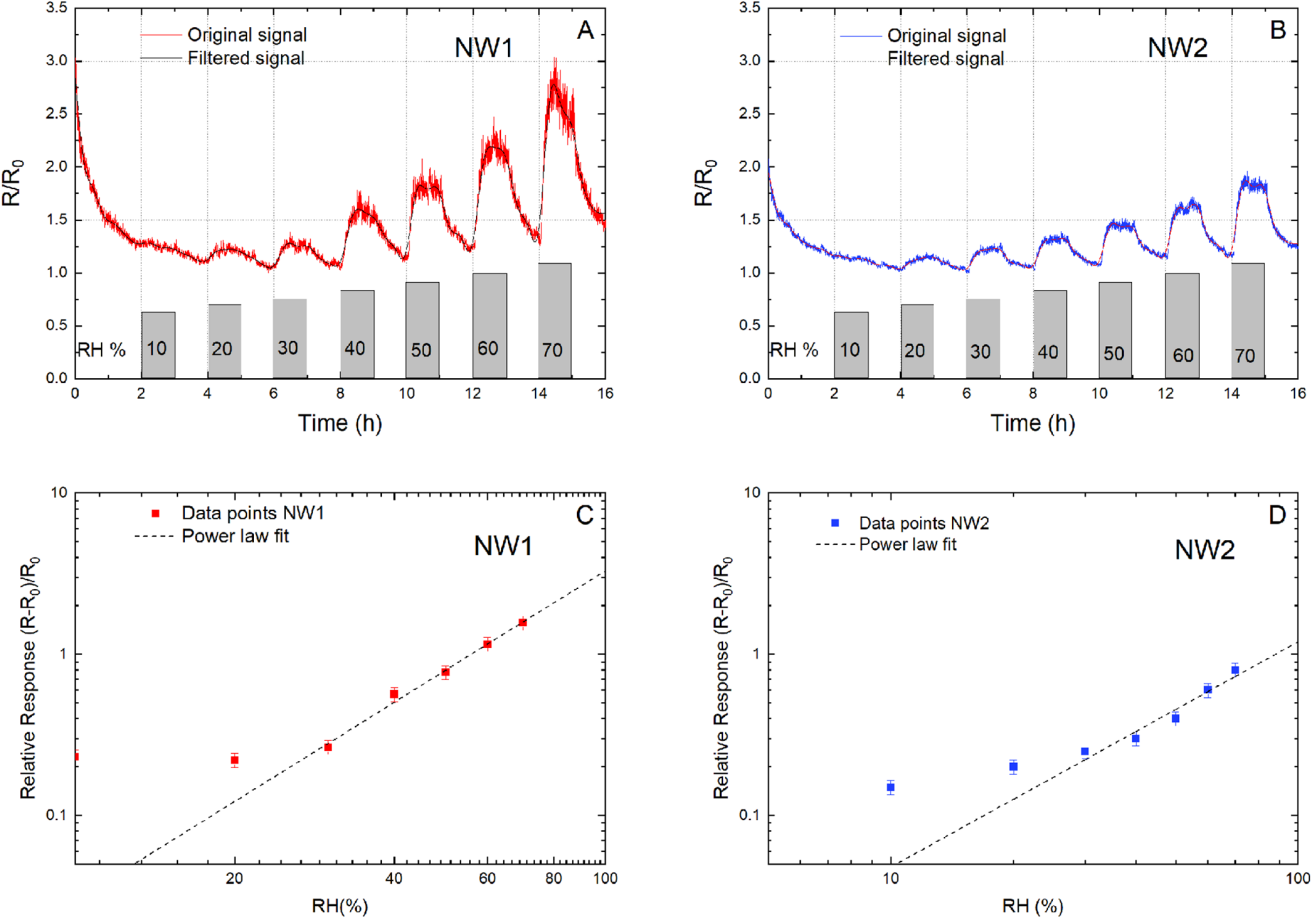
Dynamic variation of the NW twin resistance normalized to the initial (0% RH) value, toward changes in RH from 10% to 70%. Variations of relative resistance are reported for NW1 (A) and NW2 (B), together with the corresponding calibration curves for NW1 (C) and NW2 (D). Calibration curve data from 20 to 70% were fitted with a power law (*y* = 0.0026 · *x* ^2.04^ for NW1, *y* = 0.0019 · *x* ^1.39^ for NW2). Error bars indicate standard deviations. For both signals, a fifth-order digital Butterworth filter was applied with a cutoff frequency of 2.5 mHz; the filtered results are reported as thin green and red lines. This filter attenuates fluctuations in the signals with a period shorter than 400 s.

During each sensing cycle, we observed an increase in device resistance upon exposure to humidity, followed by a subsequent drop in resistance to the baseline value during the recovery period (dry air flushing), with the maximum resistance value increasing with the RH value for each cycle. The NWs were sensitive to RH in concentrations down to 20%. Prior to reaching a relative humidity (RH) of 30%, the NW twins exhibited a nearly identical resistance trend. However, as the RH exceeded 40%, a deviation was observed. For the sake of comparison, we define relative response (RR) as (*R* – *R*_0_)/*R*_0_, where *R* is the steady-state resistance in gas and *R*_0_ is the steady-state resistance in air. Specifically, the relative response of NW1 began to amplify compared to that of NW2. At an RH of 70%, the differential in relative response reaches its maximum value (approximately 1.4-fold). The electrical noise associated with NW1 also increases at higher RH. The noise analysis will be addressed in the discussion section.

Before starting the measurements and the actual test, we repeated several cycles of different relative humidity flows into the chamber to ensure the same starting conditions for each device and test. After the last cycle, we waited two h to reach the dry air condition. During this transient period, R/R_0_ decreases, and the device stabilizes to the baseline signal.

#### NO_2_ Detection

In Figure 4, we present the results of the sensing tests of the twin NW devices against NO_2_ for concentrations ranging from 2 to 9 ppm in 1 ppm increments.

**Figure 4:**
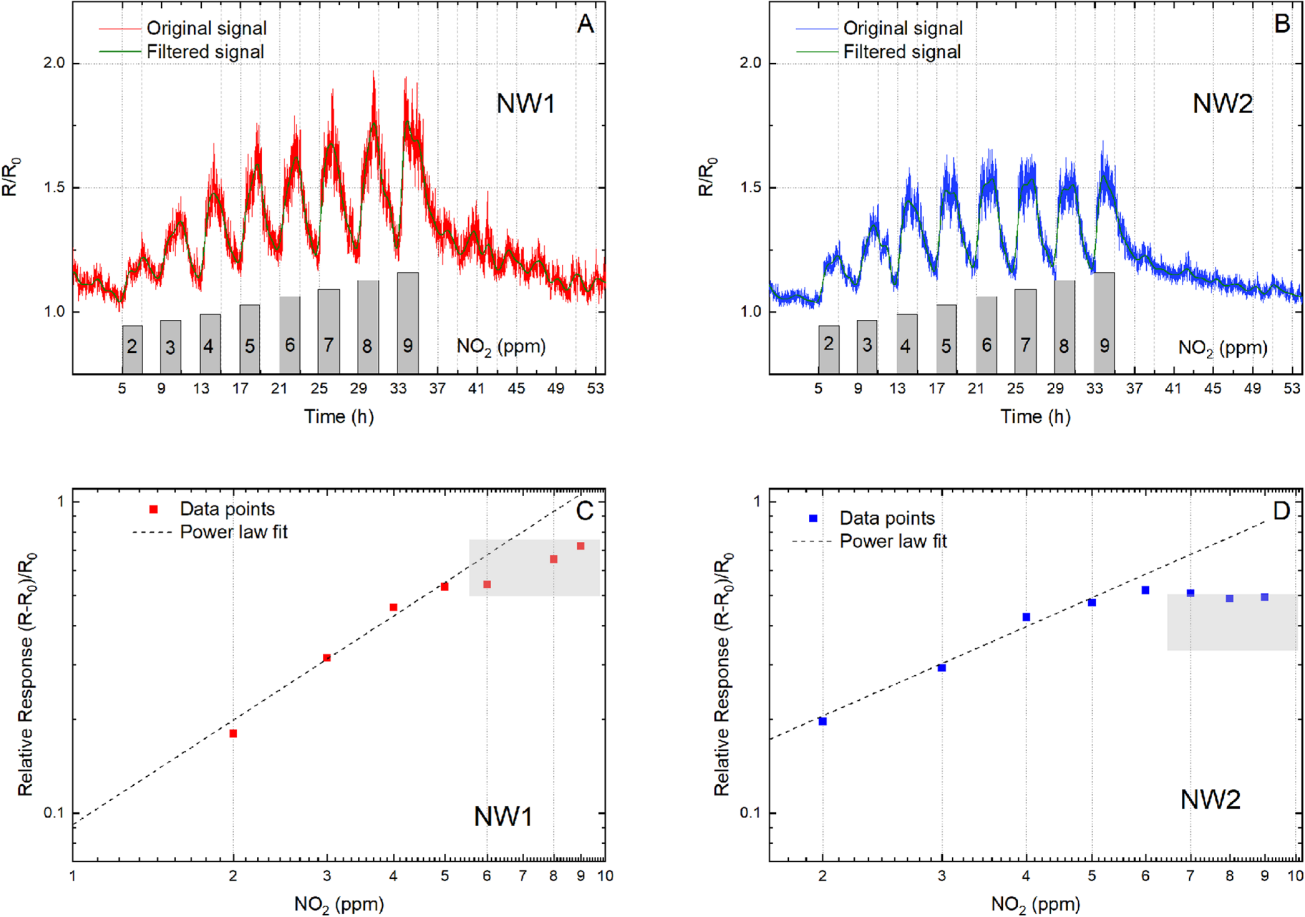
Dynamic response of the NW twins versus changes in NO_2_ from 2 to 9 ppm. Variations of relative resistance are reported for NW1 (A) and NW2 (B), along with their corresponding calibration curves for NW1 (C) and NW2 (D). For both signals, a fifth-order digital Butterworth filter was applied with a cutoff frequency of 1/900 Hz, and the results are shown as thin green lines in panels a and b. This filter attenuates fluctuations in the signals with a period shorter than 900 s.

To perform the NO_2_ sensing tests, we employed a testing cycle that comprised a 2-h exposure period during which the sensors were exposed to an input airflow with a specific and constant NO_2_ concentration, followed by a 2-h recovery period during which the samples were allowed to recover to their baseline resistance in dry air. As expected, NW twin devices exhibited sensitivity to NO_2_ even at the lowest tested concentrations. However, we observed that NW1 displayed a higher response to NO_2_ than NW2 when concentrations exceeded 4 ppm. Furthermore, both NW1 and NW2 exhibited saturation behavior at concentrations higher than 5 ppm.

During each sensory cycle, we noted a transient increase in the device resistance upon being subjected to humidity and NO_2_. This increase was followed by a decrease toward baseline resistance levels during the recovery phase, which involved dry air flushing. Notably, the InAs NW twins were able to desorb NO_2_ molecules solely through air flushing. This does not occur for NWs with different compositions, such as metal oxide NWs, which necessitate high-operating temperatures or UV-driven desorption under ambient conditions for the removal of gas molecules from their surfaces.^27^ Remarkably, slight differences occurred in the responses and similarly in the signal fluctuations of the InAs NW twin devices, and a noise analysis of the experimental data sets was carried out in order to elucidate the nature and the sources of such differences.

#### Discussion of the Response Times

We notice that quite large response times are characteristic of single nanowire-based devices made from different materials, not only III–V semiconductor compounds but also metal oxides.^23^ We test our InAs nanowire sensors in synthetic air, thus setting up operating conditions potentially relevant for real-life applications. However, this might increase the response time because of the synthetic air-nanowire surface interaction, the room temperature operation, and the use of individual nanowires. Indeed, a single nano-object can be regarded as an ultralocal probe, extremely sensitive to any change in the surrounding environment, virtually down to processes involving a few molecules, and the achievement of an equilibrium spatial distribution of molecules surrounding the nano-object can be an extremely slow process. For instance, a recent study by some of us on the electrical response of InAs nanowire transistors surrounded by charged nanoparticles suggests that up to several hundred minutes may be necessary before equilibrium is reached and a stable electrical current is achieved in the nanowire device.^45^

In principle, the twin nanodevice architecture reported in this work should allow for gate control of the electrical signal by operating the devices as field-effect transistors, applying a voltage bias to the silicon substrate, and exploiting the silicon oxide as a dielectric. However, in this scenario, even larger response times can be expected due to the hysteretic behavior of transconductance, which is typically observed at room temperature and in nonvacuum environments.

### Signal Fluctuation Analyses

In each measurement, the NW twin devices generate a pair of voltage signals that in the ideal case of identical devices should also be identical. While from a bird’s-eye view, similar trends can be identified in the signals measured in the two nanowires, a closer look reveals that each pair of measured signals displays clear differences between the two individual voltage traces of each NW, both in terms of signal intensity and signal-to-noise ratio. In order to quantitatively address the similarity of different series of data sets measured simultaneously in NW twin devices, we applied the correlation analysis method and calculated autocorrelation (AC) and crosscorrelation (CC) functions for all the experimental signal pairs. The goal was to understand the source of the signal fluctuations; to this aim, we carried out a careful analysis of the noise and its time dependence, both for individual NWs and by checking the temporal correlations among the measurement fluctuations in the NW twin devices.

For this analysis, we considered the extended set of non-normalized data shown in Figure 5, reported as resistance (*R*, panel (a)) or conductance (*S*, panel (b)) and covering the full measurement time interval, which exceeds 50 hours. In both panels, orange lines represent biexponential fits calculated for the different chamber gas insertion/removal periods. These data sets include those already reported, as renormalized resistance, in panels (a) and (b) of Figures 3 and 4.

**Figure 5:**
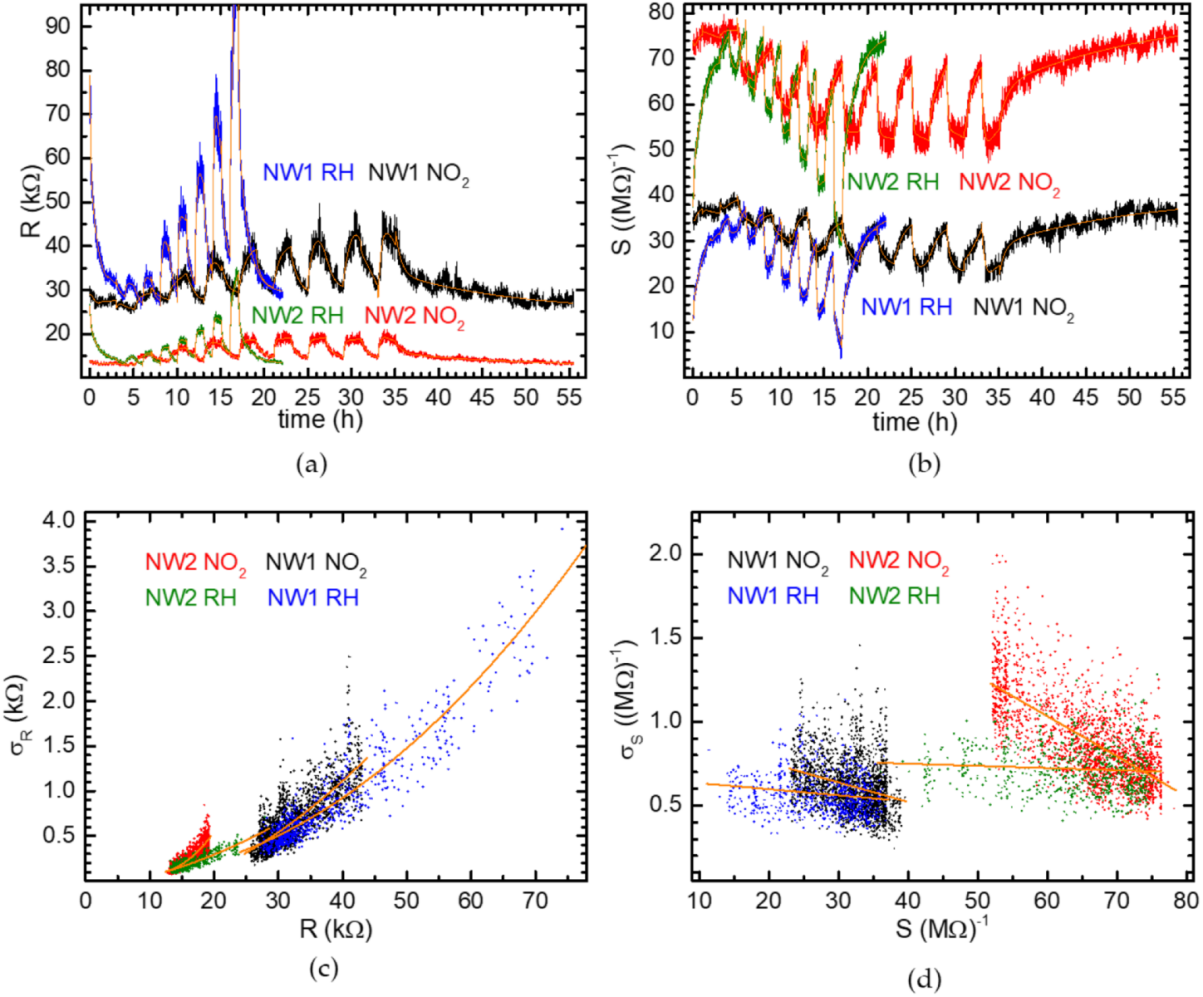
(a) Electrical resistance, *R*, and (b) conductance, *S*, as a function of time for NW twin devices exposed to RH and NO_2_. The data are the same as the ones reported in Figures 2a,b and 3a,b. Orange lines are biexponential fits. For the composition of the gas flow at different times, see Figures 3 and 4; there is an additional gas insertion phase at RH of 80% between 16 and 17 h in the RH case, but those data have not been used in any reported analysis. (c) Standard deviation of resistance and (d) conductance, extracted from the fit residuals, as a function of the fitted value of *R* and *S*, respectively. Orange lines are parabolic fits in (c) and linear fits in (d).

It can observed that the higher the resistance *R* (i.e., the measured voltage with nominally constant current), the higher the noise. Moreover, higher resistances are measured at higher concentrations of contaminants. By considering instead the conductance $S=\frac{1}{R}$ of the NWs as a function of time (Figure 5b), the noise seems more constant. This behavior is quantitatively analyzed in panels (c) and (d) of Figure 5, showing the standard deviations of the signals as a function of the signal intensities. To generate these graphs, the time dependence of *R* and *S* was fitted, within each chamber gas insertion/removal period of time, with a biexponential-decay-with-offset function (orange lines in Figure 5a,b), and each point in panels (c) and (d) represents the quadratic average of the fit residuals within a window of 40 points (corresponding to a 3.3 min time window) centered every 20 points within each chamber gas insertion/removal period. As expected, while the standard deviation σ_R_ exhibits a strong dependence on *R*, the dependence of σ_S_ upon *S* is definitely less pronounced. Besides, with the lowest amounts of RH and NO_2_, signals and their standard deviations tend to similar values for each NW (highest *S* and lowest *R* for each combination of NW and contaminant). At such low amounts of the analytes, σ_S_ is similar for the two NWs, just slightly higher for NW2. The signal-to-noise ratio (SNR) is larger for NW2, which has a starting resistance *R*_min_ (higher starting conductivity *S*_max_) smaller than that of NW1, as reported in Table 1. When increasing the concentration of the contaminants, in all measurements, the SNR decreases from its starting values SNR_0_ down to the lowest measured values SNR_high_ reported in Table 1.

**Table 1:** SNR Estimates at Low Contaminant Concentrations (SNR_0_) and at the Highest Used Ones (SNR_high_)­^a^ ^b^

Sample	contaminant	*R*_min_ (kΩ)	*S*_max_ (MΩ)^–1^	SNR_0_	*R*_max_	*S*_min_ (MΩ)^–1^	SNR_high_
NW1	RH	28	36	67	71	14	23
NW1	NO_2_	26	39	73	43	23	32
NW2	RH	13	76	109	24	42	56
NW2	NO_2_	13	76	119	19	52	42

a The SNR estimates have been calculated by considering the ratio between the conductivity *S* and its standard deviation σ_S_ as approximated by the fits in Figure 4d at the values of *S* reported in the column *S*_max_ and *S*_min_, respectively; also, the corresponding *R*_min_ and *R*_max_ are shown.

b The column “contaminant” reports the contaminant used in the corresponding measurement.

Signal fluctuations can stem from two phenomena: the change in the local concentration of the analyte and intrinsic dynamics in the sensor behavior. The latter may trivially account for the device response time or be related to the occurrence of metastable configurations in the NW-gas molecule system, changing the electrostatic environment surrounding the NW surface. The two NWs are so close to each other that they can virtually be considered as occupying the same location on the fabrication substrate. Therefore, any change in the local gas composition or concentration should impact the electrical transport in both NWs. Consistently, the main electrical transport features measured in both NWs display a very similar overall time dependence. In more detail, during the chamber gas insertion step, signal variation occurs with a time constant of around 0.1 h (six min) for both NWs in the case of RH, followed by a slower drift, especially for NW1. For NO_2_, the time constant increases to a value between 0.13 h and 0.26 h (8 and 16 min) for the higher concentrations, or seemingly up to 40 min for the lowest tested NO_2_ concentration (NW2 exhibits the slowest response). In all cases, the measured time constants are consistent with the time interval (approximately 10 min) necessary for changing the gas inside the measurement chamber (volume around one L, used gas flow of 0.1 L/min at standard temperature and pressure). Instead, during the chamber gas removal steps, the observed signal dynamics was overall significantly slower. In particular, in the initial phase of the gas removal process of the chamber, monoexponential fits return time constants between 0.3 and 0.8 h for RH, and around 1 h for NO_2_. In the longer gas removal phase, the fits return even longer time constants: about 7 h for NO_2_ for both NWs, and about 2 h (1.4 h) for NW1 (NW2) exposed to RH. Tentatively, such slow dynamics could be ascribed to the desorption of gas molecules previously trapped at the NW surfaces or to the degassing from the inner surface of the chamber.

The amplitudes of the observed fluctuations in *S* are relatively similar across all the investigated combinations of NWs and gas species. For this reason, we focused on the electrical conductivity experimental signal data sets to perform the analysis, which led to the estimation of autocorrelation and crosscorrelation functions of the measured signals. These functions allow us to visualize any fast time dependence occurring in a signal by addressing the characteristic times of the fluctuations and are calculated according to the following expression:

$$G_{ab}\left(\tau \right)=\left\langle \left(S_{a}\left(t\right)-\overset{―}{S_{a}}\left(t\right)\right)\left(S_{b}\left(t+\tau \right)-\overset{―}{S_{b}}\left(t+\tau \right)\right)\right\rangle$$

where the subscripts *a* and *b* label NW1 and/or NW2, the angular brackets indicate the average over time *t*, *S*_*a*,*b*_ represents the two signals, and *G*_*ab*_ represents the crosscorrelation (CC) between *S*_*a*_ and *S*_*b*_ or the autocorrelation (AC) function *G*_*aa*_ if *a* is equal to *b*. τ indicates the time lag (or interval) and represents the independent variable. For a stationary process, $\overset{―}{S_{a}}$ is taken as the time-independent average of *S*_*a*_. In our case, in order to correct the impact of the very slow variations, we assume $\overset{―}{S_{a}}$ as given by the fitted curves shown in Figure 5b. In the case of environmental fluctuations, the two NWs composing the NW twin devices are close enough to experience exactly the same fluctuations. Assuming that the two twin sensors are nominally identical, the conductance fluctuations observed in each nanodevice should also be identical. In this framework, the occurrence of a specific feature in the curve *G*_*aa*_ as a function of lag time but not in the *G*_*ab*_ (τ) curve indicates that signal fluctuations are not of environmental origin but instead arise from a cause that is local to each NW (e.g., minor morphological differences between the NW twin devices).

Figure 6 reports the AC and CC curves calculated for the two single NW-based devices labeled NW1 and NW2 operating under exposure to RH and NO_2_ gases. Both AC and CC curves have been calculated within each chamber gas insertion/removal time intervals (or some fractions) and then averaged. Figure 6a,b presents the CC curves together with the AC ones calculated for the entire measurement times, displaying the same data sets with a linear (6 (a)) or a logarithmic (6 (b)) scale for the independent variable, the lag time τ in hours. Notably, for low τ, the CC curves for both gases display significantly lower values compared to the corresponding AC ones. This indicates that the fluctuations experimentally observed in the conductance of the two devices very likely do not originate from fluctuations in the environment but rather from device-specific features. The autocorrelation curves for NO_2_ are stronger and longer-lasting compared to their counterparts for RH. Besides, the AC curves extracted for NW1 and NW2 decay with a very similar trend in experiments involving variations of both RH and NO_2_. Exponential fits return an average decay time for RH in the range of 0.022–0.027 h (1–2 min) and for NO_2_ an average decay time of about 0.085 h (5 min). In a biexponential fit, the two components are 0.004–0.005 h (15–20 s) and 0.035–0.055 h (2–3 min) for RH, while for NO_2_, they are 0.009–0.012 h (0.5–0.75 min) and 0.1–0.14 h (6–8.5 min).

**Figure 6:**
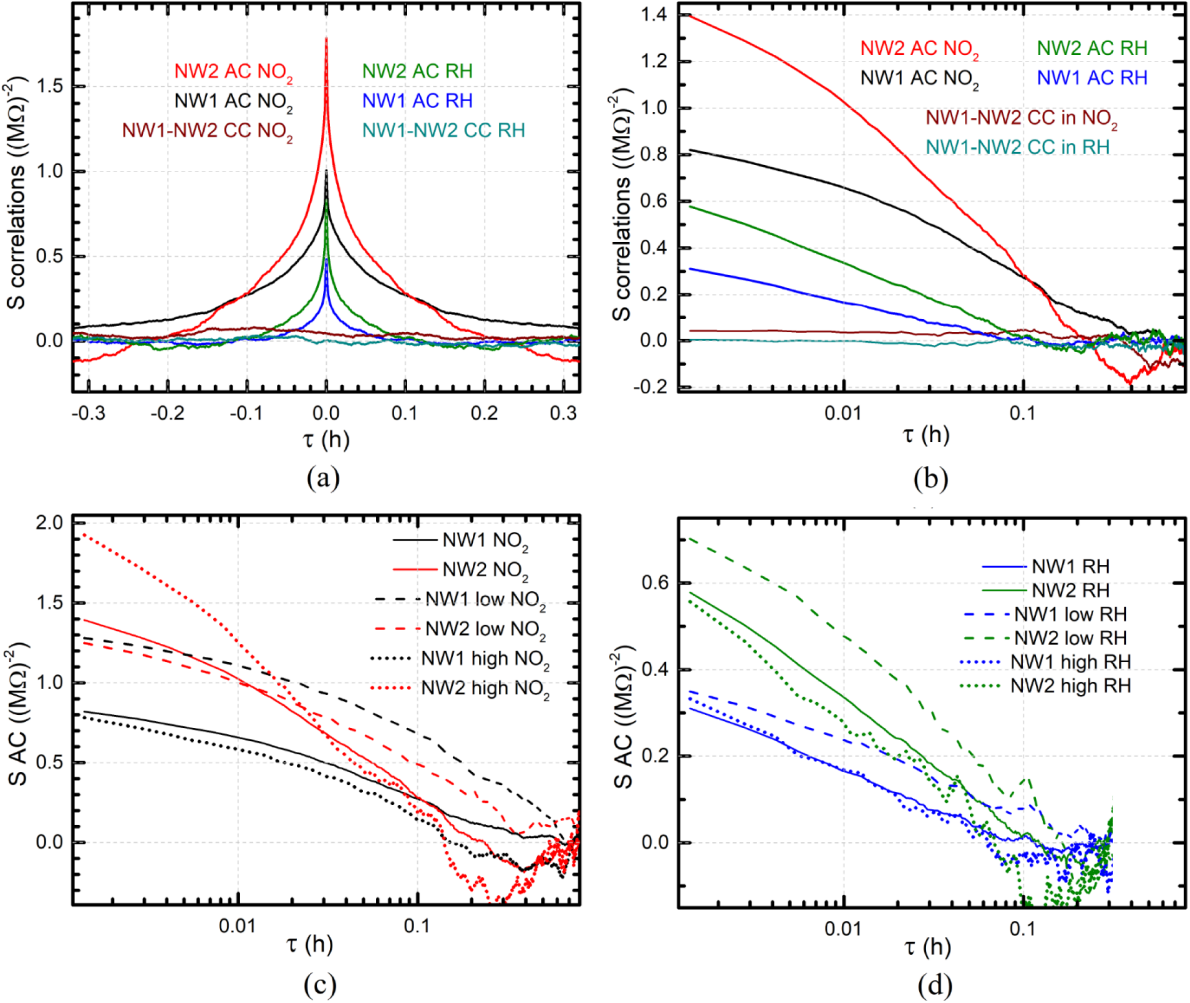
AC and CC curves for an InAs NW-based twin sensor and two different gases: (a) a linear plot and (b) a semilogaritmic plot. (c) AC curves for the two NW devices operating under NO_2_ exposure; ″high NO_2_″ labels AC curves accounting for multiple time intervals 13.5 h–15 h, 17.5 h–19 h, 21.5 h–23 h, 25.5 h–27 h, 29.5 h–31 h, and 33.5 h–35 h; ″low NO_2_″ times longer than 37.27 h. (d) AC curves for the two NW devices operating under humidity exposure; high RH indicates curves calculated from the conductance in the time intervals 6.55 h–7.03 h, 8.56 h–9.03 h, 10.57 h–11.04 h, 12.58 h–13.05 h, and 14.58 h–15.06 h; ″low RH″ accounts for times longer than 17.79 h.

If the conductance fluctuations depend on the presence of gas molecules, then they might also depend on the gas concentration. To clarify these aspects, we compared the AC curves of both NWs for two different concentrations of NO_2_ (Figure 6c) and RH (Figure 6d). In panel 6c, the label ″low NO_2_″ refers to AC functions calculated starting from the signal region corresponding to the last longer gas removal phase (time longer than 37.27 h), corresponding to an estimated concentration below, at most, 3 ppm, while the label ″high NO_2_″ refers to a signal region at or close to the plateau characteristic of the gas insertion phases, corresponding to concentrations above or around 4 ppm; solid lines correspond to the curves averaged over the whole experiment, as reported also in panel 5b, and are shown here for comparison. In panel 6d, the label ″low RH″ refers to times above 17.79 h, corresponding to about 30% RH, while ″high RH″ refers to a signal region at or close to the plateau during the gas insertion phases, corresponding to RH values above or around 30% RH; solid lines correspond to the curves averaged over the whole experiment, as reported also in panel 5b, and are shown here for comparison. Interestingly, the AC curves for NO_2_ display slower decay with respect to RH, even in the low-contaminant case. This implies that the explored NO_2_ concentration was never low enough to have a negligible impact on the noise, making it very difficult to discriminate between two different noise sources, namely electrical measurements and fluctuations in the interactions between NWs and gas molecules.

We also notice a few slow macroscopic signal fluctuations occurring, especially in the tail of the last NO_2_ gas removal phase (more evident for NW1; see Figure 5d). Similar slow fluctuations can be observed in all fit residuals for the last gas removal phase, with a typical duration of about one h for NO_2_ and half an hour for RH, i.e., close to the times characteristic of signal changes in the removal phases. Such fluctuations occur for both NWs and are uncorrelated, revealing that their origin cannot be ascribed to differences in the environment of the twin NWs. Moreover, the occurrence of these fluctuations might play a role in the onset of the nonmonotonic behavior and the negative values observed in the correlation curves at the latest lag times, as visible in Figure 6. Keeping in mind the fluctuation-dissipation theorem, we tentatively rationalize these experimental outcomes as follows. The slow dynamics in the removal phases is likely ascribable to a slow response of the NWs upon removal of the gas molecules, most probably because of the slow desorption of the gas molecules from the NW surfaces, especially in the NW regions close to the ohmic contacts. This scenario indicates a possible route for enhancing the response time of InAs NW-based gas sensors, namely, increasing the operating temperature of the NW, especially when the gas concentration is relatively low. However, the possible improvement of the gas sensing performance of InAs nanodevices, or even the use of different and more performant nanoscale systems as demonstrators, would be compatible with the overall methodology and approach developed in this work.”

## Conclusions

We reported the combined experimental measurement and signal correlation analysis of the electrical response of InAs nanowire-based twin devices used to probe different gases in synthetic air. We engineered pairs of nanowire-based nominally identical sensors (twin sensors) and simultaneously measured their response upon exposure to relative humidity or NO_2_ flux, resorting to a custom readout interface designed and realized in-house. Autocorrelation and crosscorrelation analyses were performed on simultaneously measured pairs of current signals, suggesting that changes at nanoscopic levels (i.e., local to each nanodevice), related to the nanostructure and device morphology, represent the major source of the lack of cross-correlation in the InAs nanowire-based twin devices, thus ruling out a prominent role of inhomogeneities in the chemical environment. Very importantly, while the use of different nanoscale material systems or nanodevice architectures might change the type and level of the input/output signals, it does not require a change in the methodology and preserves the validity of the approach. Indeed, the latter can be summarized as follows: (i) Selection of the type of nanomaterial suitable for the targeted conductometric sensing application, taking into account the feasibility of isolating individual nanocrystals and fabricating two-terminal devices, preferably with ohmic contacts; (ii) measurement of the size/morphology distribution of the starting nanomaterials; (iii) fabrication of two nominally identical nanodevices at a distance over which one might reasonably expect to have the same gaseous environment; (iv) simultaneous measurement of the response of the twin devices upon exposure to the gas; (v) analysis of the electrical signal level and noise of each device; (vi) correlation of the differences observed in the measurements, the individual nanoobjects, and the nanodevice architectures. Notably, points (i)–(vi) define a sequence of actions that can be regarded as a general prescription for testing the ability of any electrically conducting nanoscale object to enable simultaneous gas detection functionalities.

Considering the novelty and pioneering nature of the approach, a double validation against at least two different gases was required. In this validation, similar signal levels were generated by each nanodevice of the twin sensors upon exposure to different concentrations of the two tested gases. Our experimental environment is fully controlled and monitored, preventing spurious effects that might arise due to the co-presence of different gases in the measurement chamber: however, in a real environment, the methodology proposed in our work retains its validity, provided an additional step of chemical functionalization of the nanomaterial is performed to achieve selectivity toward specific gas molecules.

Potential applications of the technology developed in this work include the use of our methodology as a protocol for identifying batches or even classes of conducting nanomaterials that are suitable or not suitable for sensing applications. In addition, by increasing the number of nominally identical nanosensors or the distance between pairs of nanosensors, our approach might enable the measurement of the spatial inhomogeneity of the gas environment.

## References

[1] Milone A., Monteduro A. G., Rizzato S., Leo A., Di Natale C., Kim S. S., Maruccio G. (2023). Advances in Materials and Technologies
for Gas Sensing from Environmental and Food Monitoring to Breath Analysis. Adv. Sustainable Syst..

[2] Patolsky F., Lieber C. M. (2005). Nanowire nanosensors. Mater.
Today.

[3] He B., Morrow T. J., Keating C. D. (2008). Nanowire sensors for multiplexed
detection of biomolecules. Curr. Opin. Chem.
Biol..

[4] Rocci M., Demontis V., Prete D., Ercolani D., Sorba L., Beltram F., Pennelli G., Roddaro S., Rossella F. (2018). Suspended
InAs Nanowire-Based Devices for Thermal Conductivity Measurement Using
the 3ω Method. J. Mater. Eng. Perform..

[5] Demontis V., Zannier V., Sorba L., Rossella F. (2021). Surface Nano-Patterning
for the Bottom-Up Growth of III-V Semiconductor Nanowire Ordered Arrays. Nanomater.

[6] Demontis V., Rocci M., Donarelli M., Maiti R., Zannier V., Beltram F., Sorba L., Roddaro S., Rossella F., Baratto C. (2019). Conductometric Sensing
with Individual InAs Nanowires. Sens.

[7] Zagaglia L., Demontis V., Rossella F., Floris F. (2021). Semiconductor nanowire
arrays for optical sensing: A numerical insight on the impact of array
periodicity and density. Nanotechnology.

[8] Zagaglia L., Demontis V., Rossella F., Floris F. (2022). Particle swarm optimization
of GaAs-AlGaAS nanowire photonic crystals as two-dimensional diffraction
gratings for light trapping. Nano Express.

[9] Floris F., Fornasari L., Marini A., Bellani V., Banfi F., Roddaro S., Ercolani D., Rocci M., Beltram F., Cecchini M., Sorba L., Rossella F. (2017). Self-Assembled InAs
Nanowires as Optical Reflectors. Nanomater.

[10] Yang W., Xie M., Zhang X., Sun X., Zhou C., Chang Y., Zhang H., Duan X. (2021). Multifunctional
Soft Robotic Finger
Based on a Nanoscale Flexible Temperature-Pressure Tactile Sensor
for Material Recognition. ACS Appl. Mater. Interface.

[11] Keum K., Cho S. S., Jo J.-W., Park S. K., Kim Y.-H. (2022). Mechanically
robust textile-based strain and pressure multimodal sensors using
metal nanowire/polymer conducting fibers. iScience.

[12] Nikolaeva A. V., Kondratev V. M., Kadinskaya S. A., Kolesina D. E., Zubov F. I., Anikina M. A., Dvoretckaia L. N., Lendyashova V. V., Gridchin V. O., Monastyrenko A. O., Krasnikov D. V., Nasibulin A. G., Kochetkov F. M., Bolshakov A. D. (2025). ZnO nanowire-based
flexible sensors for pressure and temperature monitoring. Mater. Sci. Semicond. Process..

[13] Kondratev V. M., Vyacheslavova E. A., Shugabaev T., Kirilenko D. A., Kuznetsov A., Kadinskaya S. A., Shomakhov Z. V., Baranov A. I., Nalimova S. S., Moshnikov V. A., Gudovskikh A. S., Bolshakov A. D. (2023). Si Nanowire-Based Schottky Sensors
for Selective Sensing of NH3 and HCl via Impedance Spectroscopy. ACS Appl. Nano Mater..

[14] Bârsan N. (2011). Transduction
in Semiconducting Metal Oxide Based Gas Sensors - Implications of
the Conduction Mechanism. Procedia Eng..

[15] Zhang Y., Kolmakov A., Chretien S., Metiu H., Moskovits M. (2004). Control of
Catalytic Reactions at the Surface of a Metal Oxide Nanowire by Manipulating
Electron Density Inside It. Nano Lett..

[16] Mirzaei A., Lee J.-H., Majhi S. M., Weber M., Bechelany M., Kim H. W., Kim S. S. (2019). Resistive
gas sensors based on metal-oxide
nanowires. J. Appl. Phys..

[17] Mirzaei A., Leonardi S. G., Neri G. (2016). Detection
of hazardous volatile organic
compounds (VOCs) by metal oxide nanostructures-based gas sensors:
A review. Ceram. Int..

[18] Baratto C., Golovanova V., Faglia G., Hakola H., Niemi T., Tkachenko N., Nazarchurk B., Golovanov V. (2020). On the alignment
of ZnO nanowires by Langmuir - Blodgett technique for sensing application. Appl. Surf. Sci..

[19] Comini E., Baratto C., Faglia G., Ferroni M., Vomiero A., Sberveglieri G. (2009). Quasi-one dimensional metal oxide
semiconductors: Preparation,
characterization and application as chemical sensors. Prog. Mater. Sci..

[20] Tan H. M., Hung C. M., Ngoc T. M., Nguyen H., Hoa N. D., Duy N. V., Hieu N. V. (2017). Novel Self-Heated
Gas Sensors Using
on-Chip Networked Nanowires with Ultralow Power Consumption. ACS Appl. Mater. Interfaces.

[21] Baratto C. (2018). Growth and
properties of ZnO nanorods by RF-sputtering for detection of toxic
gases. RSC Adv..

[22] Kolmakov A. (2008). Some recent
trends in the fabrication, functionalisation and characterisation
of metal oxide nanowire gas sensors. Int. J.
Nanotechnol..

[23] Hernández-Ramírez F., Tarancón A., Casals O., Arbiol J., Romano-Rodríguez A., Morante J. R. (2007). High response and stability in CO and humidity measures
using a single SnO2 nanowire. Sens. Actuators,
B.

[24] Meng G., Zhuge F., Nagashima K., Nakao A., Kanai M., He Y., Boudot M., Takahashi T., Uchida K., Yanagida T. (2016). Nanoscale
Thermal Management of Single SnO2 Nanowire: Pico-Joule Energy Consumed
Molecule Sensor. ACS Sens..

[25] Donarelli M., Ferroni M., Ponzoni A., Rigoni F., Zappa D., Baratto C., Faglia G., Comini E., Sberveglieri G. (2016). Single Metal
Oxide Nanowire devices for Ammonia and Other Gases Detection in Humid
Atmosphere. Procedia Eng..

[26] Baratto C., Kumar R., Faglia G., Vojisavljević K., Malič B. (2015). p-Type copper aluminum oxide thin
films for gas-sensing
applications. Sens. Actuators, B.

[27] Zhang D., Liu Z., Li C., Tang T., Liu X., Han S., Lei B., Zhou C. (2004). Detection of NO2 down to ppb Levels Using Individual
and Multiple In2O3 Nanowire Devices. Nano Lett..

[28] Du J., Liang D., Tang H., Gao X. P. (2009). InAs Nanowire Transistors
as Gas Sensor and the Response Mechanism. Nano
Lett..

[29] Lynall D., Tseng A. C., Nair S. V., Savelyev I. G., Blumin M., Wang S., Wang Z. M., Ruda H. E. (2020). Nonlinear Chemical
Sensitivity Enhancement of Nanowires in the Ultralow Concentration
Regime. ACS Nano.

[30] Offermans P., Crego-Calama M., Brongersma S. H. (2010). Gas Detection with Vertical InAs
Nanowire Arrays. Nano Lett..

[31] Zhang X., Fu M., Li X., Shi T., Ning Z., Wang X., Yang T., Chen Q. (2015). Study on the
response of InAs nanowire
transistors to H2O and NO2. Sens. Actuators,
B.

[32] Ullah A. R., Joyce H. J., Tan H. H., Jagadish C., Micolich A. P. (2017). The influence
of atmosphere on the performance of pure-phase WZ and ZB InAs nanowire
transistors. Nanotechnol.

[33] Prete D., Dimaggio E., Demontis V., Zannier V., Rodriguez-Douton M. J., Guazzelli L., Beltram F., Sorba L., Pennelli G., Rossella F. (2021). Electrostatic Control of the Thermoelectric
Figure
of Merit in Ion-Gated Nanotransistors. Adv.
Funct. Mater..

[34] Prades J. D., Jimenez-Diaz R., Hernandez-Ramirez F., Barth S., Cirera A., Romano-Rodriguez A., Mathur S., Morante J. R. (2008). Ultralow power consumption
gas sensors based on self-heated individual nanowires. Appl. Phys. Lett..

[35] Chikkadi K., Muoth M., Maiwald V., Roman C., Hierold C. (2013). Ultra-low
power operation of self-heated, suspended carbon nanotube gas sensors. Appl. Phys. Lett..

[36] https://www.ti.com/lit/ds/symlink/lm334.pdf (Accessed on 17 October 2023).

[37] https://www.analog.com/media/en/technical-documentation/data-sheets/AD8655_8656.pdf (Accessed on 17 October 2023).

[38] https://www.ti.com/lit/ds/symlink/ads1220.pdf (Accessed on 17 October 2023).

[39] Endres H.-E., Jander H. D., Göttler W. (1995). A test system for gas sensors. Sens. Actuators, B.

[40] Voliani V., Ricci F., Luin S., Beltram F. (2012). Peptidic coating
for
gold nanospheres multifunctionalizable with photostable and photolabile
moieties. J. Mater. Chem..

[41] Adler J., Parmryd I. (2010). Quantifying colocalization by correlation: The Pearson
correlation coefficient is superior to the Mander‘s overlap
coefficient. Cytometry, Part A.

[42] Greiner, A. ; Korvink, J. G. Analysis of noise and fluctuations in micromachined devices Micromachined Devices and Components V SPIE 1999 104–112

[43] Di
Carlo L., Zhang Y., McClure D. T., Marcus C. M., Pfeiffer L. N., West K. W. (2006). System for measuring auto- and cross
correlation of current noise at low temperatures. Rev. Sci. Instrum..

[44] Ries J., Schwille P. (2012). Fluorescence correlation
spectroscopy. BioEssays.

[45] Prete D., Colosimo A., Demontis V., Medda L., Zannier V., Bellucci L., Tozzini V., Sorba L., Beltram F., Pisignano D. (2023). Heat-Driven Iontronic Nanotransistors. Adv.
Sci..

